# Exploring the Microfluidic Production of Biomimetic Hybrid Nanoparticles and Their Pharmaceutical Applications

**DOI:** 10.3390/pharmaceutics15071953

**Published:** 2023-07-14

**Authors:** Dafina Fondaj, Ilaria Arduino, Angela Assunta Lopedota, Nunzio Denora, Rosa Maria Iacobazzi

**Affiliations:** Department of Pharmacy-Pharmaceutical Sciences, University of Bari, 70125 Bari, Italy; dafina.fondaj@uniba.it (D.F.); ilaria.arduino@uniba.it (I.A.); angelaassunta.lopedota@uniba.it (A.A.L.); nunzio.denora@uniba.it (N.D.)

**Keywords:** biomimetic, nanoparticles, hybrids, microfluidics

## Abstract

Nanomedicines have made remarkable advances in recent years, addressing the limitations of traditional therapy and treatment methods. Due to their improved drug solubility, stability, precise delivery, and ability to target specific sites, nanoparticle-based drug delivery systems have emerged as highly promising solutions. The successful interaction of nanoparticles with biological systems, on the other hand, is dependent on their intentional surface engineering. As a result, biomimetic nanoparticles have been developed as novel drug carriers. In-depth knowledge of various biomimetic nanoparticles, their applications, and the methods used for their formulation, with emphasis on the microfluidic production technique, is provided in this review. Microfluidics has emerged as one of the most promising approaches for precise control, high reproducibility, scalability, waste reduction, and faster production times in the preparation of biomimetic nanoparticles. Significant advancements in personalized medicine can be achieved by harnessing the benefits of biomimetic nanoparticles and leveraging microfluidic technology, offering enhanced functionality and biocompatibility.

## 1. Introduction

Over the course of billions of years, nature has continuously adapted and evolved, creating highly efficient and enduring solutions to overcome the challenges of survival. Humans have proven that they can create innovative and sustainable technologies that can help address some of the most challenging problems facing our planet today [1,2,3,4,5]. This approach has also been applied to the development of new methods of disease treatment and diagnosis. Replicating the sensitive natural mechanisms found in the human body is a challenging task. As a result, scientists have turned to nature for inspiration rather than solely relying on novel solutions.

Biomimetic nanotechnology focuses on understanding the fundamental aspects of living systems and adapting their qualities for human use, particularly at the nanometer scale; this shift in approach has paved the way for personalized treatment strategies improving the functionality of nanoparticles and streamlining their development process [6]. In fact, traditional therapy and treatment methods have several limitations, such as difficulties in delivering active chemicals, poor water solubility, low oral bioavailability, and unfavorable side effects that outweigh the therapeutic benefits.

### 1.1. Nano Delivery Systems in Therapy and Diagnosis

In response to the concerns regarding conventional drug delivery systems, research teams are progressively focusing on the development and application of nanoparticles (NPs) as drug carriers, including liposomes, polymer NPs, solid lipid NPs, hybrid NPs, and biomimetic NPs [7,8,9,10,11,12,13,14]. In comparison with free medicinal molecules, NP-based delivery methods have numerous significant benefits [15,16,17], such as improved drug solubility, stability, large drug payloads, precise delivery, and the ability to be administered via various routes, most notably through parenteral administration [18,19]. Given the significance of NPs’ interactions with biological systems, it is widely recognized that the intentional engineering of their surfaces is a vital aspect of the entire design process of NPs. In addition to reducing nonspecific NP uptake, the inclusion of targeting mechanisms can assist increase effectiveness even further by increasing preferential accumulation at a specific area of interest [20,21]. The incorporation of polyethylene glycol (PEG) onto the surface of a particle has been the most popular standard in the process of creating NPs. This method led to the formation of stealth NPs with less interaction with their surroundings, which enabled a longer duration of blood circulation [22,23]. Although PEG is successful in reducing nonspecific interactions in complex media, there have been reports of immunological reactions and the presence of antibodies against PEG that might affect the performance of such NPs across several administrations [24,25]. Recently, also as an answer to this problem, known as the PEG dilemma, biomimetic NPs are increasingly being investigated. In conclusion, the development of NPs as drug carriers offers promising advancements in addressing the drawbacks of traditional drug delivery systems due to their tailored surfaces and enhanced delivery capabilities.

### 1.2. Biomimetic Hybrid Drug Delivery Systems

Biomimetic NPs are novel structures that mimic the properties of biological systems found in nature. These nanocarriers are typically created through the combination of materials such as lipids, polymers, and metals with cellular components to mimic the functions of natural biomolecules. The principle of developing biomimetic NPs is based on harnessing the efficiency and specificity of biological and natural systems for a variety of applications, ranging from drug delivery to imaging, sensing, and catalysis processes, to provide enhanced functionality and improved biocompatibility [26,27,28]. The main reasons why NPs should be made biomimetic NPs are as follows: to evade the immune system and immunological reactions, to improve the stability and longevity of the NPs themselves, to prevent nonspecific uptake by healthy cells, and to improve the targeting of the desired cells [29,30]. The essential goal is to create nanostructures with surfaces that enable them to be ignored by everything except their target, a challenge that has so far proved extremely difficult. In fact, regardless of how intriguing an experimental drug or material seems to be in vitro, effective biointerfacing is essential for successful translation in vivo [31]. Researchers have explored a variety of biomimetic NPs to overcome all the issues described above, such as membrane-derived molecules and subunits, membrane-bound biomacromolecules, cell-membrane-based nanostructures, and vesicular structures [32]. A significant number of studies have been carried out and continue to be conducted concerning the efficacy and feasibility of employing biomimetic NPs for the treatment of illnesses, such as infectious diseases [33] and inflammation [27], as well as novel strategies for tumor immunotherapy in which biomimetic NPs present a promising strategy [34]. In spite of ongoing studies across research groups, biomimetic NPs, particularly those coated with cell membranes, have made significant progress by entering clinical trials, demonstrating a promising strategy for the development and success of biomimetic NPs as drug delivery systems [35]. In this review, an overview of the literature about biomimetic NPs and their production methods is included, with a focus on the microfluidic technique as an innovative strategy for the efficient and tuned formulation of biomimetic NPs, characterized by scalability, high monodispersity, rapid and continuous production, and versatility in accommodating various materials [36,37].

## 2. Classification, Diversity, and Application of Biomimetic Hybrid NPs

Recently cell-membrane-camouflaged NPs have emerged as a biomimetic platform for drug delivery. Cell membranes of interest can be extracted and coated onto NPs or used as building blocks to form NPs on their own or with other polymeric entities. Cell membranes of erythrocytes, platelets, leukocytes, and tumor cells have been exploited to engineer NPs with surface characteristics of source cells. In addition, cell membrane camouflage does not require the use of labor-intensive complicated bioconjugation methods, making itself an appealing approach for the generation of biofunctionalized nanoparticles. Ultimately, biomimetic hybrid NPs, in terms of structure, can be mainly classified into two categories:

(a) Coated biomimetic hybrid NPs, made of molecules such as lipids, polymers, and proteins, or synthetic materials such as silica or gold, coated by a layer of biological material. The coating creates a protective layer around the core and may additionally enhance stability, control release kinetics, improve biocompatibility, and facilitate targeting [38,39].
(b) Fused biomimetic hybrid NPs, which entail the direct integration of biological components into the NPs’ surface structure. The biological component becomes a part of the NPs’ structure either physically or chemically by fusing with the main components of NPs. This integration provides unique biological properties and functionalities, such as specific targeting, enzymatic activity, or cell recognition [40,41].

**Figure 1:**
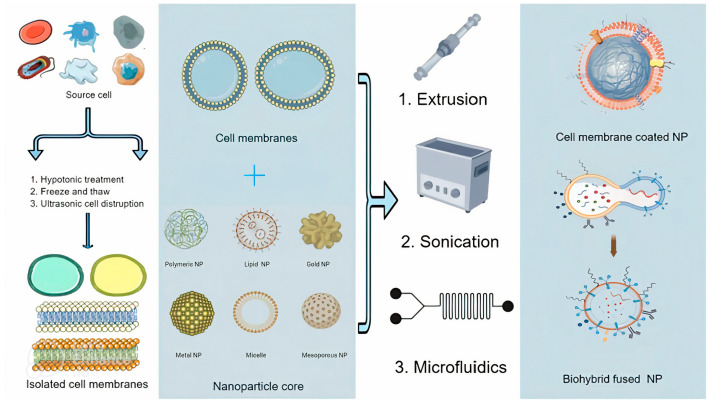
Scheme of different biomimetic NP production methods. This figure was partly generated using Servier Medical Art, provided by Servier, licensed under a Creative Commons Attribution 3.0 unported license.

Regarding their production, both types of biomimetic NPs could be developed covering nanomaterials or engineering them, with a layer of parts of natural cell membranes generated from several cell types (Figure 1). In addition, biomimetic NPs can be created using a variety of techniques, including molecular imprinting, emulsion techniques, self-assembly methods, and co-precipitation methods. Recently, microfluidics has emerged as a powerful technique for biomimetic NP production by enabling the development of NPs with desired properties by supplying precise control over fluid flow and reaction conditions.

### 2.1. Coated Biomimetic Hybrid NPs

**Figure 2:**
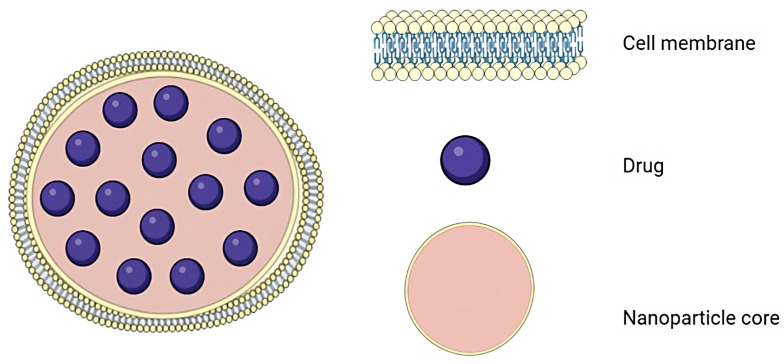
Structure of a cell-membrane-coated NP. Figure was partly generated using Servier Medical Art, provided by Servier, licensed under a Creative Commons Attribution 3.0 unported license, and adapted from the attached reference [46].

Cell-membrane-coated NPs (CMNPs) are an example of biomimetic nanotechnology that encapsulates NPs with a layer of cell membrane derived from living cells (Figure 2). To construct core–shell NP structures, a distinct number of cell membranes have been involved, such as red blood cells, white blood cells, platelets, specific cancer cells, etc. [42,43,44]. CMNPs have received significant attention in a variety of fields, including drug delivery, imaging, and biomedical research [45].

**Figure 3:**
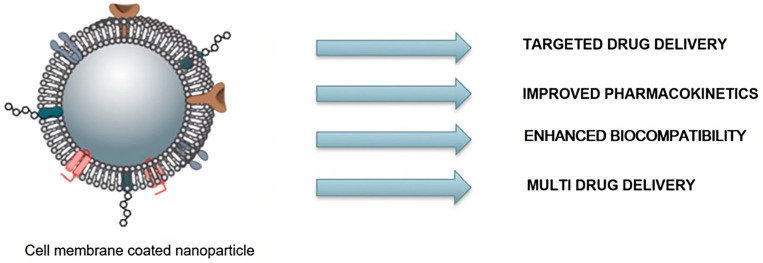
Benefits of cell-membrane-coated NPs in drug delivery. The figure was partly generated using Servier Medical Art, provided by Servier, licensed under a Creative Commons Attribution 3.0 unported license.

These emerging NPs offer several benefits as novel drug delivery systems, with improved pharmacokinetics, enhanced biocompatibility, and as a possible multidrug delivery system (Figure 3).

#### 2.1.1. Red-Blood-Cell-Membrane-Coated NPs

The cell membrane coating technique was initially described in 2011, in which researchers used entire cell membranes as a material for NP coating [47]. This approach was initially shown using red blood cells (RBCs) as the source of membrane material. RBCs, the most prevalent type of cell in human blood, are highly specialized cells with distinguishing characteristics, such as size, shape, mechanical flexibility, and chemical composition, all of which are tailored for superior biological performance. The use of RBCs to create biomimetic nanocarriers exploits the advantages of such cells, including immune evasion, high circulation time, decreased reticuloendothelial (RES) clearance, the avoidance of serum protein adsorption, and resistance to complement reactions, which all add up to erythrocyte membranes being one of the best options of coating material for increasing medication biocompatibility [48,49]. Thus, the coating of NP cores with RBC membranes to produce RBC-membrane-coated NPs (RBCMNPs) enables the minimization of toxicity, the enhancing of stability, and preventing the immune system from identifying the nanopreparations. These RBCMNPs are designed to carry therapeutic materials, such as proteins, nucleic acids, and small-molecule medications, for a variety of applications, such as autoimmune diseases, tumor imaging, photothermal therapy, photodynamic chemotherapy, antibacterial vaccine, etc. [50,51,52,53,54,55]. Several of these nanoplatforms are already in significant clinical studies to treat a wide range of diseases, including cancer and enzyme deficiencies [56,57]. In a study presented by a group of researchers, RBCMNPs were created with a paclitaxel (PTX)-loaded polymeric core and a hydrophilic RBC vesicle coating. The elimination half-life of the RBCMNPs was 32.8 h, which is 5.8 times longer than the elimination half-life of the parental polymeric NPs (i.e., 5.6 h) [58]. Another advantage of NPs coated with RBC membrane is the capability to deliver cargo while evading an immunogenicity reaction, in comparison with bare NPs [59]. Another example is the loading of doxorubicin (DOX) into poly (lactic acid), PLA, cores and subsequent coating with RBC membrane [60]. RBC membrane can also be used to coat NPs loaded with a magnetic component, to offer magnetic-guided distribution of NPs to a specific place. In the first example of such a system, chemotherapeutics, such as PTX and DOX, were encapsulated together with iron oxide nanocrystals in O-carboxymethyl-chitosan NPs using a double emulsion method [61]. Other research found that a DOX-loaded RBCMNP formulation improved survival in a mouse model of lymphoma when compared to equal dosages of free medicine. In this study, it was demonstrated that the RBCMNPs were typically safe and had no myelosuppressive impact, whereas the free medication produced a significant reduction in various immune cell subtypes. Moreover, zero anti-RBC antibodies were found in mouse serum after multiple RBCMNP doses, showing that the NPs generated neither acute nor long-term immunological reactions [62]. Based on the abovementioned studies, they can offer the easiest path to translation, and there is the possibility to employ type-matched RBCs as membrane sources to perfect biocompatibility for widespread clinical applications.

**Figure 4:**
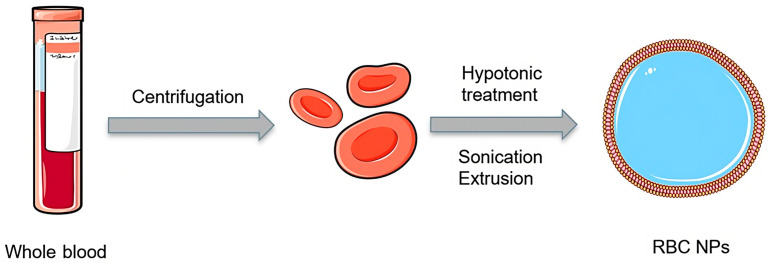
Formulation of red blood cell NPs. Figure was partly generated using Servier Medical Art, provided by Servier, licensed under a Creative Commons Attribution 3.0 unported license. Adapted with permission from [68]. Copyright {2018} American Chemical Society.

Preparing the cellular membranes and particle cores separately, and then proceeding with the coating process of the NPs offers a novel level of engineering for biomimetic NPs with higher functionalities [63]. Deriving the membrane from RBCs and membrane particle coating are two essential steps in the effective and common production of RBCMNPs. To make RBCs vesicles, two procedures are combined: hypotonic treatment and sequential extrusion; then, the coating of NPs with RBCs occurs [64,65,66]. Typically, prepared NPs and derived red blood cell vesicles are merged using mechanical extrusion (Figure 4); RBCMNPs can generally be acquired using different methodologies, such as the co-extrusion method, microfluidic electroporation method, or cell-membrane-templated polymerization [67].

Considering their potential and unique qualities as novel drug delivery systems, RBCMNPs are being investigated for a wide range of applications. They are being studied and researched for various applications and have reached the milestone of going through clinical trials, as was previously mentioned. Despite supplying numerous benefits as a novel drug delivery system, there are several parameters to consider when using RBC membranes as a coating, such as the source of the blood. Since this technology has great potential for personalized therapy, requiring blood from clinics will be an emerging requirement. Additional hurdles include batch-to-batch reproducibility, mass production for individual patients requiring specific blood types, post-manufacturing storage conditions, and regulatory approval, which has been difficult despite the proven promise of these nanoformulations [69].

#### 2.1.2. Platelet-Cell-Membrane-Coated NPs

**Figure 5:**
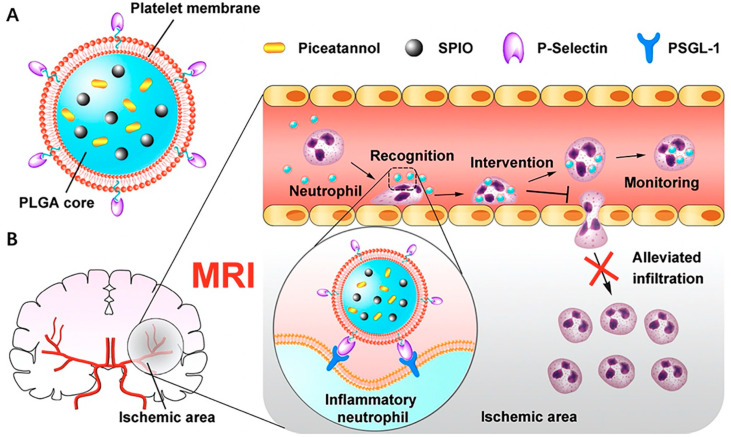
(**A**) Structure of platelet membrane coated PLGA NPs; (**B**) Schematic presentation of therapeutic mechanism, licensed under a Creative Commons Attribution 3.0 unported license, adapted with permission from [79]. Copyright {2021} American Chemical Society.

Platelet cells (PCs) are natural blood components characterized by a prolonged circulation time [70], and have attracted a great deal of attention. Their primary purpose is to maintain hemostasis, and since they are naturally drawn to areas of vascular damage to initiate a cascade that leads to clot formation, initiating the healing process, they also perform a range of different activities and have been linked to the pathophysiology of several disorders, including cancer, atherosclerosis, and bacterial infections; therefore, as natural substances in the bloodstream, they do not only assist in homeostasis but are also engaged in several processes associated with disease development [71]. Moreover, PCs can resist macrophage absorption due to the presence of additional receptors on their surface and have been studied for their impact on tumors owing to their interactions with receptors found in tumor cell membranes [72]. PCs’ structures and capabilities allow them to create novel opportunities for their use as innovative nanoplatforms and drug carriers. NPs coated with platelet cell membrane (PCMNPs) are promising as biomimetic therapeutics and diagnostic tools [73]. PC membranes contain a variety of surface components with diverse abilities to interact with biological systems. For example, signal regulatory protein α (SIRP α) is an immunoglobulin that occurs mainly in neurons and myeloid cells. The activation of SIRP α in phagocytic cells via interaction with CD47 on PCs reduces phagocytosis [74]. Consequently, this mechanism also aids in evading the rapid clearance of PCMNPs. Moreover, PCMNPs can also experience prolonged circulation time. Due to all of the mechanisms and benefits mentioned above, PCMNPs have found application as drug delivery systems; functionalizing the nanoparticles with PC membranes enables drug-loaded NPs to bind to specific targets and can be guided to the specific disease sites while reducing the off-target properties. The use of PCMNPs in the treatment of thrombotic diseases has shown great potential due to their ability to encapsulate anticoagulant drugs and mimic the natural properties of platelets to provide a targeted treatment. PCMNPs can also be functionalized with imaging and contrast agents and be utilized for the detection and diagnosis of a certain disease, such as cancer or cardiovascular disorders [75,76,77]. Chi et al. developed a drug delivery system for the treatment of lung cancer by coating those NPs of sufficient size (98.2 nm) to benefit from the EPR effect, allowing them to accumulate in tumor tissue. The NPs’ slow release of the loaded drug, docetaxel, ensured long-term therapeutic efficacy. The PCM coating added additional advantages, such as avoiding immune recognition and clearance by the RES, resulting in prolonged bloodstream circulation, and accumulated specifically in lung tumors. Experiments in vitro revealed that PCM/PLGA/DTX NPs successfully impeded the growth of lung cancer cells. In vivo studies on mice with A549-cell-derived tumors confirmed the NPs’ significant inhibitory effect on tumor growth. Importantly, the PCM/PLGA/DTX NPs were less toxic than free docetaxel. This implies that the NPs’ formulation improves the drug’s therapeutic index, making it safer and more tolerable for patients [78]. Tang et al. creatively created a platelet-membrane-coated PLGA nanoplatform with superparamagnetic iron oxide and piceatannol-loaded nanoparticles (PCNPs) in response to these discoveries. Through the recognition of P-selectin (a platelet protein) and PSGL-1 (a neutrophil protein), the coated platelet membranes, in their study, facilitated binding between nanoparticles and neutrophils. Piceatannol, which was released by the internalized nanoconstructs, reduced the amount of neutrophil infiltration in the ischemic areas of the brain. In contrast to the group given PLGA nanoparticles containing piceatannol and superparamagnetic iron oxide, PTNPs significantly reduced the infarct volume of mice by about 26.2% (Figure 5) [79].

In another research study, PCMNPs were produced using a cell membrane cloaking approach, and their ultimate size was around 15 nm greater than plain NPs. In addition, as compared to bare NPs and RBCMNPs, PCMNPs showed improved in vivo permeability and retention. PCMNPs can traverse circulation and selectively distribute to damaged tissues rather than healthy tissues [80]. Wei et al. coated an NP core with PCM, which can interact with the endothelium, foam cells, and collagen. These effects have been shown to be distinctive to PCMNPs. The developed NPs were capable of efficiently localizing well developed atherosclerotic plaque as well as artery regions prone to plaque formation. The authors used magnetic resonance imaging to detect the PCMNPs’ effect, which was demonstrated using an atherosclerosis animal model [81]. The coating of NPs with PCM has led to the creation of many other mimicking systems based on platelets that target many health issues, such as trauma [82], vascular diseases [76], inflammation [83], and cancer [84]. Another group of scientists directly employed PCs to carry doxorubicin (DOX) to treat lymphoma, and they discovered that DOX loaded in PCs targeted cancer cells successfully via the phenomenon known as tumor-cell-induced PC aggregation, “TCIPA”; therefore, as a result, cancer cell growth inhibition was increased while DOX cardiotoxicity was significantly reduced [85,86]. Regardless of the potential of PCMNP preparations as a therapeutic option for the treatment of a variety of illnesses, several relevant aspects must be addressed prior to them being ready for clinical use, such as comprehending the process of the interaction between specific cells and PCs, manufacturing and storage issues, safety, and pharmacokinetic properties [87].

#### 2.1.3. Leukocyte-Cell-Membrane-Coated NPs

**Figure 6:**
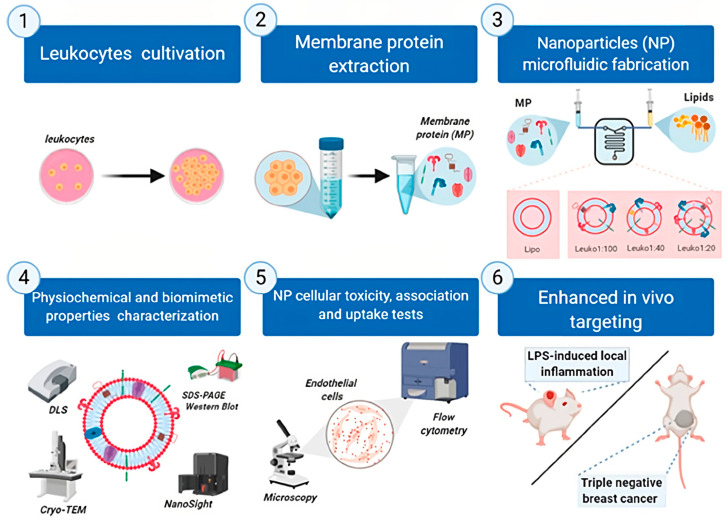
Graphical view of leukocyte NPs, the production process via the microfluidic technique, and biomimetic properties characterization licensed under a Creative Commons Attribution 3.0 unported license, adapted with permission from [92]. Copyright {2021} American Chemical Society.

White blood cells (WBCs) are essential immune cells that play a role in many serious diseases, including cancer, infections, and inflammatory disorders. Due to WBCs’ multifaceted and dynamic functions, scientists have begun to investigate their potential for the development of novel therapeutics. A potential strategy is the creation of WBC-membrane-coated NPs (WBCMNPs). To create these NPs, the plasma membranes of specific WBCs, such as macrophages, neutrophils, T cells, and natural killer cells, are used to coat synthetic NP cores. This approach combines the distinct properties of WBCs with the versatility and functionality of synthetic NPs, opening new possibilities for targeted drug delivery and immunotherapy [43]. Leukocyte-coated NPs, also known as leukosome NPs (LCMNPs), are a form of drug delivery method in which NPs are coated with leukocyte membranes. The complex biological recognition mechanisms constitute an important barrier in advancing this type of NP formulation from the state of lab production to the bedside. NPs, for example, can be identified as foreign compounds by macrophages and be eliminated from an organism without reaching their target. This, along with numerous other obstacles, such as targeted administration and tissue penetration, is particularly important for the clinical translation of nanomedicines and represents issues that require a solution [88,89]. However, LCMNPs can replicate the natural capabilities of leukocytes, such as a low degree of protein absorption and the capacity to target as well as adhere to specific tissues or cells inside the body that enable more precise and effective therapeutic agent delivery to different parts of the body. Among all leukocytes, in recent times, where cell-membrane-based NP formulations have become a common method of formulating nanocarriers, macrophages have attracted great attention in being developed as NP coatings [90]. It has been demonstrated that, as compared to ordinary NPs, macrophage-membrane-coated NPs (MCMNPs) may efficiently transport anticancer medicines to specific regions of the body and achieve significant accumulation duration with a controlled release impact, all this because they can move easily across extravascular tissues and blood vessels. A team of researchers created RAW 264.7 macrophage-cell-membrane-coated mesoporous silica NPs (MSNs) for tumor-specific doxorubicin distribution (DoxMPCM-camouflaged MSN). MSNs possess homogeneous cylindrical pores and a wide surface area, which allow for a high loading capacity. The outcomes of in vivo imaging and anticancer assays showed that these biomimetic NPs extended blood circulating time and increased treatment effectiveness in a subcutaneous 4T1 xenograft model [91]. Zinger et al. used microfluidic production to create biomimetic leukocyte NPs. The formulated NPs were tuned to change their efficiency by increasing the lipid–protein ratio, and their microfluidic production allowed them to create nanoparticles with the ability to enhance the treatment of inflammatory-based conditions via these biomimetic NPs (Figure 6) [92].

T cells, on the other hand, are composed of several subsets, including CD4+ T cells (helper T cells), CD8+ T cells (cytotoxic T cells), and regulatory T cells [93]. Zhang et al. presented a unique technique with which to neutralize HIV viruses, since they were able to fabricate CD4+-T-cell-membrane-coated PLGA NPs by effectively extracting the cell membranes, hence proving that utilizing T cell subsets for medical applications may improve the precision of molecular recognition as well as the efficacy of disease control [94]. Proteins that are originally found on the plasma membrane play a critical role in leukocyte biology; therefore, by producing this type of coated NP, the biological properties of the source cell, such as specific site targeting and cellular self-recognition, as well as proteins typically found on the membrane (e.g., CD47, LFA-1, and MAC-1) are faithfully transferred and preserved onto the NPs [95]. Despite the benefits listed above, there is still a lot of room to investigate feasible and scalable LCMNPs. Numerous aspects need to be investigated further to speed up clinical translation. Quality control is a significant problem for LCMNP coating, and standard techniques should be created. Additionally, because the challenges that these biomimetic NPs present when delivered in vivo are not completely known, multidisciplinary collaborations in chemistry, biology, and medicine need to be reinforced to improve these LCMNPs. Even though this strategy is new, preliminary research suggests that it offers considerable potential for the treatment of a variety of disorders.

Leukocyte-cell-membrane-coated nanoparticles can be used for a variety of exciting medical and biotechnological applications. These nanoparticles can modulate the immune system by utilizing leukocyte characteristics, allowing for targeted drug delivery and focused infection as well as inflammation treatment. Additionally, they have the potential to be used for imaging, diagnosis, tissue regeneration, and repair. To make their design and implementation into clinical practice as effective as possible, additional research is needed. Leukocyte-membrane-coated nanoparticles have the potential to have a significant impact on several industries by offering novel approaches to disease diagnosis, treatment, and regenerative medicine.

#### 2.1.4. Cancer-Cell-Membrane-Coated NPs

**Figure 7:**
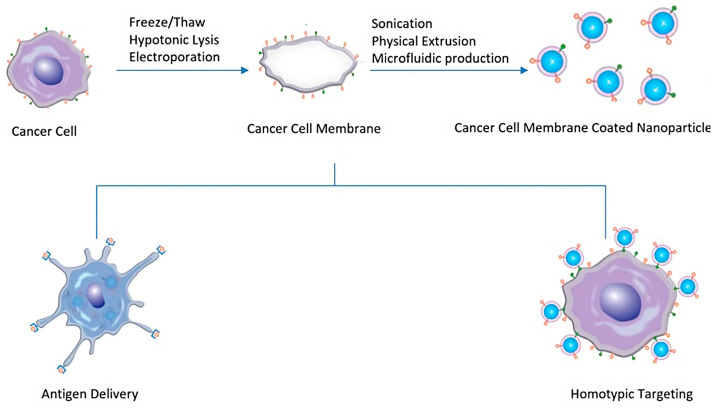
Scheme of the production of cancer-cell-membrane-coated NPs (CCMNPs) licensed under a Creative Commons Attribution 3.0 unported license, adapted with permission from [106].

Cancer is one of the most challenging diseases to diagnose and treat, as well as a worldwide public health problem that requires innovative methods to provide effective treatment. While there have been considerable breakthroughs in cancer therapy over the years, researchers and physicians still face several hurdles in the battle against cancer. Traditional cancer treatment procedures include the surgical excision of tumors, followed or preceded by intensive chemotherapy and localized radiation; however, if tumors are unresectable or have metastasized, chemotherapy continues to be the primary treatment option for attempting to reduce the growth and spread of cancer. Despite being the most often used clinical treatment, cytotoxic therapeutics are incapable of targeting only cancer cells and being precise even after systemic treatment [96,97]. Just a small percentage of medications accumulate in tumors and metastatic lesions before being removed from the body or infiltrating non-targeted tissues [98]. As a result, undesirable side effects for healthy tissues restrict the amount of the drug that may be given, therefore reducing effectiveness [99]. In recent years, NP technology has enabled several advances in cancer treatment, ranging from better effectiveness in cancer medication delivery to the higher immunogenicity of cancer vaccines [100]. This novel strategy allows for the development of nanovehicles capable of delivering single or many therapeutic cargo as well as contrast agents to tumors for enhanced therapy and diagnosis. NPs have the potential to improve medication delivery, phototherapy, immunization, and immunotherapy by overcoming immune evasion mechanisms, enhancing the effectiveness of immunotherapies, and promoting antitumor immune responses, in addition to imaging by serving as agents in various modalities, aiding in cancer detection and monitoring treatment responses [101,102]. The tumor microenvironment is distinguished by leaky vasculature and inadequate lymphatic drainage; an increased EPR effect is used by all systemically delivered nanocarriers to passively accumulate and be maintained in tumor tissue [103,104]. In the field of biomimetic NP-based therapy and diagnosis, an emblematic role is played by cancer cells as sources of cell membrane to be used for coating synthetic NPs. In fact, cancer cells, unlike most other membrane donors, are simple to cultivate in enormous quantities in vitro; they also have the unique capacity to self-target homologous cells, a phenomenon also known as homotypic targeting. This distinct capacity transfers to cancer-cell-membrane-coated NPs (CCMNPs), which retain the potential to target cancer cells homotypically [105] (Figure 7). Several NP core designs may be used to build CCMNPs depending on the designated application. Regardless of core material, the fundamental criteria are that the NPs have a negative zeta potential. Due to the electrostatic repulsion between the NP surface and negative extracellular membrane components, an appropriate membrane orientation around the NP is created. In general, three processes follow the production of CCMNPs: membrane extraction from source cells, nanoparticle core manufacturing, and the fusing of membranes and nanoparticle cores to create core–shell membrane-coated NPs. CCMNPs could be produced by three main strategies, involving extrusion, sonication, and the microfluidic technique, as novel approaches to producing biomimetic NPs (Figure 7) [106].

Several research studies have shown that encapsulating DOX in a biomimetic nanosystem, specifically in CCMNPs, is far superior to free drug delivery in terms of a significantly improved antitumor efficacy by increased accumulation to the tumor site and a lower overall systemic toxicity [107]. Another aspect to be considered is that, in solid tumors, a lack of oxygen causes hypoxia, which automatically leads to drug resistance, and the efficacy of the treatment is not desirable. Thus, to overcome chemoresistance caused by tumor hypoxia, CCMNPs have recently been designed to deliver a combination of chemotherapeutics, such as the drug DOX, and the oxygen carrier hemoglobin (Hb). MCF-7 cancer cell membranes were used to coat PLGA NPs loaded with DOX and Hb to form dual drug-loaded CCMNPs. The nanoformulation demonstrated homotypic binding properties, with the parent MCF-7 cells exhibiting the highest DOX uptake when compared to the DOX uptake into other cancerous and noncancerous cells [108].

While the application of CCMNPs as a personalized cancer therapy holds great promise, there are still several challenges that need to be resolved before it can become a commercially available technology. To ensure that the membrane coatings are pure and do not contain any molecules that could promote cancer growth, stringent testing and regulatory procedures must be developed. The feasibility of developing patient-specific CCMNPs is also an important question, as developing patient-specific CCMNPs will necessarily require rigorous quality assurance and regulatory procedures.

According to several studies regarding CCMNPs, they hold great promise for a broad range of applications, the following being among them: Targeted drug delivery, which enables the NPs to deliver therapeutic agents to cancer cells while increasing the drug concentration at the tumor site and therefore improving the chemotherapy efficiency. These biomimetic NPs also offer enhanced tumor penetration and improve treatment outcomes.

#### 2.1.5. Dual-Membrane-Coated Hybrid NPs

While the single-cell membrane coating strategy has increased the use of NPs, other biological functions can also be added for unique applications. The dual-membrane coating strategy involves fusing cell membranes from two different sources and then coating a synthetic NP, giving such a biomimetic hybrid nanosystem the properties of both cell lines. Even though biological functionality is derived from various source cells, the criteria for selecting cell membranes are primarily determined by the qualities of source cells and the requirement of the disease to offer efficient treatment. Following that, erythrocyte–cancer hybrid cell membrane-camouflaged NPs (RBC/CancerMNPs), platelet–leukocyte hybrid cell membrane-camouflaged NPs (PC/LeuM-NPs), and cancer stem cell–platelet hybrid cell membrane-camouflaged NPs (CPVM-NPs) were successfully created for several applications [109,110,111,112]. This innovative and promising approach of developing dual-cell-membrane-based biomimetic hybrid NPs demonstrated the potential to increase the efficacy of existing therapies in addition to showing promising results as a potential strategy for the targeted delivery of drugs and other therapeutic agents.

**Figure 8:**
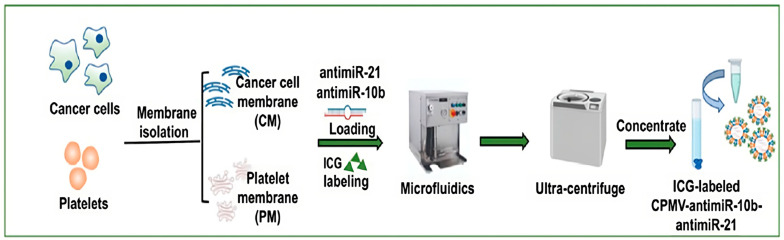
Schematic presentation of the preparation of hybrid biomimetic NPs with the microfluidic technique. Adapted and reproduced with permission [113]. Copyright© 2023 Wiley-VCH GmbH.

Particularly, cancer cell–platelet fusion membrane vesicle NPs, CPMVNPs, loaded with therapeutic microRNAs (miRNAs) have shown promise in the treatment of triple-negative breast cancer (TNBC). In this study, cell membranes extracted from breast cancer cells and platelets were used to generate CPMVs, and their formulation was conducted using the microfluidic technique, which represents a novel approach of designing and formulating biomimetic NPs (Figure 8) [113]. An in vitro analysis revealed that the CPMVNPs recognized their source cells and avoided being taken up by macrophages. Furthermore, after systemic administration in mice, the CPMVNPs demonstrated a long-lasting circulation time and site-specific deposition at triple-negative breast cancer xenografts. The delivered anti-miRNAs sensitized TNBCs to DOX, leading to an improved therapeutic reaction and survival rate.

Wang et al. created a dual-membrane hybrid biomimetic coating (RBC-B16) by fusing membrane materials derived from RBCs and melanoma cells (B16-F10) cells. This dual-membrane was used to conceal doxorubicin-loaded hollow copper sulfide NPs (DCuS-RBC-B16) NPs for melanoma combination therapy. The DCuS-(RBC-B16) NPs exhibited the inherent properties of both source cells, including in vitro self-recognition to the source cell line, prolonged circulation lifetime, and improved targeting abilities in vivo. The DOX-loaded (RBC-B16)-coated CuS NP platform demonstrated an excellent synergistic photothermal effect inherited by the conversion of CuS and chemotherapeutical effects of DOX, resulting in melanoma tumor growth inhibition of approximately 100% [114].

Jang and colleagues fused RBC membranes with MCF-7 cell membranes, and therefore created RBC–cancer cell melanin NPs to overcome the drawbacks traditional therapy offers and improve anticancer efficacy. According to the findings of this study, this dual-membrane hybrid biomimetic nanoparticle platform has shown advantageous properties, such as homotypic targeting, long circulation, good biocompatibility, controllability, and photothermal benefits, making it ideal for clinical applications [115].

#### 2.1.6. Exosome-Based Nanodrug Delivery Systems

**Figure 9:**
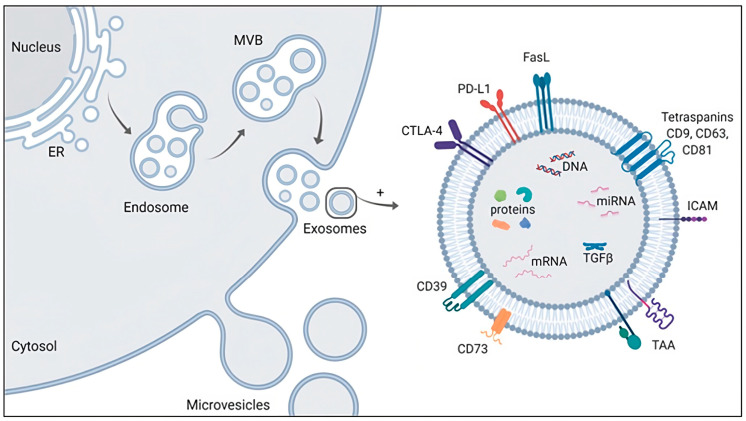
Schematic presentation of exosomes licensed under a Creative Commons Attribution 3.0 unported license, adapted with permission from [119].

Exosomes are tiny, membrane-bound vesicles produced by cells that transport a variety of biomolecules, such as proteins, nucleic acids, and lipids. Trams et al. originally reported exosomes, which were then confirmed by Johnstone et al., who found certain levels of transferrin binding onto tiny particles (exosomes), but the same binding activity was lost on parent cells [116,117]. Exosomes are created inside the cell by invaginating into endosomal membranes to form multivesicular bodies (MVBs) (Figure 9) [118].

Due to their capacity to target specific cells and tissues, exosomes have received a lot of interest in recent years as drug delivery vehicles. Researchers show that exosomes are linked to various defining aspects of cancer, such as promoting tumor angiogenesis, rebuilding the stroma to develop the tumor microenvironment, and enabling tumor growth as well as medication resistance via genetic information transfer between cancer cells [120]. Exosomes are rich in transmembrane proteins and adhesion proteins, as well as specialized protein receptors; hence, protein payloads on exosomes may be efficiently transported to and received by specified cells to cause biological responses, such as the induction and promotion of neoplasia [121]. Based on this information, exosomes have an exceptional ability to evade immune system clearance due to their possession of CD47, which is a highly expressed integrin-related transmembrane protein that works to shield cells from phagocytosis [122]. Inspired by all of these natural properties, exosomes present a great possibility to be engineered as drug delivery systems, due to their capacity to infiltrate other cells effectively with minimal immune clearance even after repeated injections, which are easily tolerated without any adverse effects, therefore allowing them to become utilized for treatment purposes (Table 1) [123,124].

Among the many possibilities as potential drug vehicles that exosomes provide, the natural affinity of tumor-derived exosomes to certain organs was recently the one that has been investigated more [131]. It was discovered that exosomes exhibited different biodistributions based on the source tumor; this process was integrin-dependent and corresponded with future metastatic locations. In another study, macrophage-derived exosomes were employed to deliver DOX specifically due to their inherent ability to target tumors [132]. Exosome-loaded carriers kill tumor cells more efficiently than conventional chemotherapeutics yet exhibit fewer cytotoxicity effects in myocardial cells and other healthy tissues [133]. Exosomes, among others, have also been used to formulate NPs. This method integrates the natural membrane characteristics of exosomes with the functionality of NPs. A group of scientists created a formulation of paclitaxel-loaded exosomes (AA-PEG-exoPTX), based on aminoethylanisamide-polyethylene glycol (AA-PEG-exports), with a high loading capacity and the ability to accumulate in lung cancer cells. The incorporation of an (AA-PEG) vector allowed for specific targeting of the sigma receptor overexpressed in these cells. The AA-PEG-exoPTX formulation demonstrated enhanced therapeutic outcomes, showing the potential of exosome-based drug delivery for enhanced anticancer therapy [134].

### 2.2. Fused Biomimetic Hybrid NPs

Fused biomimetic hybrid NPs are NPs that are designed to mimic the properties of natural living organisms and simultaneously take advantage of the properties of synthetic components to ensure greater uniformity of the chemical–physical and morphological characteristics of the nanosystem, resulting in better formulation stability. In fact, such particles are engineered to have specific properties that make them useful for a wide range of applications. A common approach to developing biomimetic hybrid NPs is to use natural biological molecules as key components, such as proteins or lipids. Researchers can take advantage of these natural materials’ distinctive attributes, such as their ability to self-assemble or interact with specific biological targets, by incorporating them into the nanoparticle. Such nanoparticles are fabricated by inserting human cell membranes into the synthetic lipid bilayer, leading to the synthesis of a core–shell structure with biomimetic properties.

**Figure 10:**
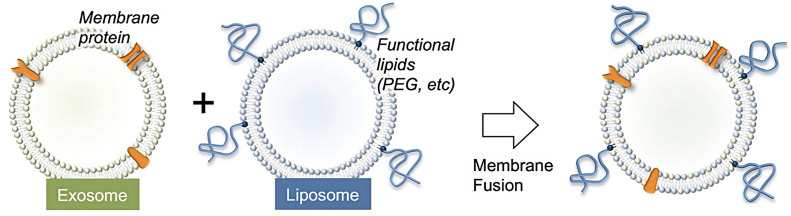
Schematic presentation of the fusion of exosomes with liposomes, creating a fused hybrid nanodelivery system licensed under a Creative Commons Attribution 3.0 unported license, adapted with permission from [137].

As fundamental units of cells, membranes are responsible for a wide range of functions, including the ability to interface and interact with the surrounding environment; a biomimetic hybrid therefore exploits this portion in its entirety, rather than isolating a single component, by combining the advantages of a naturally derived cell membrane with the synthetic nanocarrier. Each cell membrane component (lipids, proteins, and carbohydrates) performs a specific function for cell survival: lipids confer fluidity and participate in signal transduction, while proteins and carbohydrates are responsible for interactions with other proteins, other cells, or extracellular components of the outer matrix. Replicating this entire system artificially would be impossible, so researchers have employed the principles of biomimetic design to achieve the sensitivity and specificity of a single cell type [135]. Sato et al. presented a new approach to improving the performance of exosomes as drug delivery systems in their study. The researchers used the freeze–thaw method to fuse their membranes with liposomes, resulting in hybrid exosomes (Figure 10). They demonstrated that genetic modification techniques can be combined with membrane engineering methods by isolating a specific membrane protein from genetically modified cells and embedding it into exosomes. This enabled them to design fused hybrid exosome–liposome NPs with specific properties (Table 2). Cellular uptake studies on these fused hybrid exosomes revealed that, by changing the lipid composition or properties of the lipids added to the exosomes, the interactions between the exosomes and cells could be modified. This finding implies that the membrane engineering strategy presented in this study offers a novel strategy for developing exosomes as hybrid nanocarriers with controlled properties. Overall, this study demonstrates the potential of fused hybrid exosomes as advanced drug delivery systems. Exosomes can be rationally designed to improve their performance by combining genetic modification and membrane engineering techniques, opening new avenues for targeted and efficient drug delivery [136].

**Figure 11:**
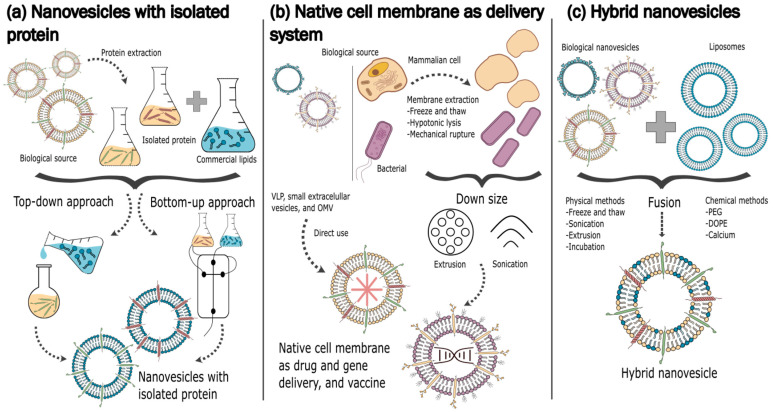
Design and production of different biomimetic nanovesicles licensed under a Creative Commons Attribution 3.0 unported license, adapted with permission from [138].

Fusing liposomes with cell membranes is a process that allows for the creation of biomimetic NPs capable of delivering a drug load while also maintaining the stability of liposomes to create a more specific formulation for medical applications. One or two cell membranes can be fused together with a synthetic liposome in this process. When final hybrid liposomes are compared to purified lipids and a single membrane protein or membrane collection, the latter improves the nanovesicle’s biomimetic property. In this case, the biomimetic NPs exhibit several distinct characteristics (Figure 11) [138].

**Figure 12:**
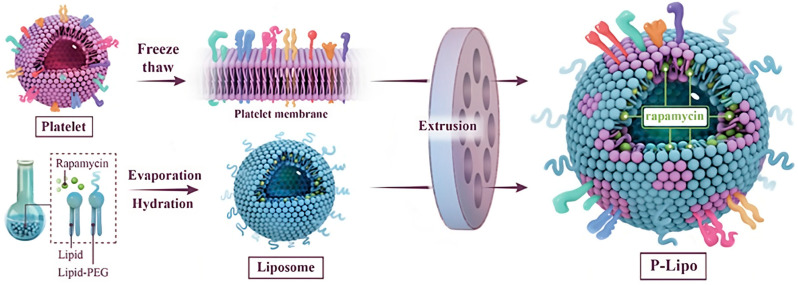
Fused hybrid biomimetic platelet membrane–liposome. Adapted and reproduced with permission from [139]. Copyright © 2023 Elsevier B.V.

In another study, biomimetic liposomes made of synthetic liposomes hybridized with platelet membranes (P-Lipo) were created, by the extrusion technique, for atherosclerosis targeting because they were inspired by the interaction of platelet membrane components with atherosclerosis plaques (Figure 12). P-Lipo effectively inhibited atherosclerosis development in all of the treatment groups without causing cytotoxic effects, using the atheroprotective drug rapamycin as the model drug. This study presented P-Lipo as an effective option for the treatment of atherosclerosis and many other diseases where platelets are implicated [139].

To evade immune surveillance, a composite cell-membrane-camouflaged biomimetic nanoplatform, namely leutusome, which is made of liposomal NPs incorporating plasma membrane components derived from both leukocytes (murine J774A.1 cells) and tumor cells (head and neck tumor cells, HN12), was formulated. Exogenous phospholipids were used as building blocks to fuse with two cell membranes to form liposomal nanoparticles. The anticancer drug paclitaxel (PTX) was used to make drug-encapsulating liposomal nanoparticles. Leutusomes resembling characteristic plasma membrane components of the two cell membranes were examined and confirmed in vitro. A xenograft mouse model of head and neck cancer was used to profile the blood clearance kinetics, biodistribution, and antitumor efficacy of the different liposomal nanoparticles. The results demonstrated that leutusomes obtained prolonged blood circulation and were most efficient when accumulated at the tumor site. Similarly, leutusome composed of membrane fractions of B16 melanoma cells and leukocytes (J774A.1) showed prominent accumulation within the B16 tumor, suggesting the generalization of the approach. Furthermore, PTX-encapsulating leutusome was found to inhibit tumor growth most potently, while not causing systemic adverse effects [140].

**Table 2:** Summary of different hybrid NPs and their therapeutic effects.

Membrane Source	Characteristic	Effect	Reference
Exosome–liposome	Targeted and efficient drug delivery	Cancer and inflammation	[136]
Platelet–liposome	No cytotoxicity effects	Treatment of atherosclerosis	[139]
Leukocyte–cancer cell	Tumor targeting, increased circulation time in vivo	Accumulation in tumor site	[140]

## 3. Production of Biomimetic Hybrid NPs

In general, compared to non-hybrid NPs and conventional treatment approaches, hybrid biomimetic NPs have demonstrated advantages as an innovative approach to developing new treatment strategies, not just for cancer but also for other diseases, as stated in this review. Comparing hybrid biomimetic systems to non-hybrid ones, they have shown superiority in loading effectiveness, release kinetics, cellular uptake, and cytotoxicity. Biomimetic hybrid NPs are a very promising therapeutic strategy, but they also have some development challenges: these nanocarriers are extremely difficult to create and synthesize, and their production requires a multistep process. Given that they are made up of numerous complex components, it may be difficult to predict how these components will interact, with the knowledge that maintaining and controlling their stability is very difficult [141].

**Figure 13:**
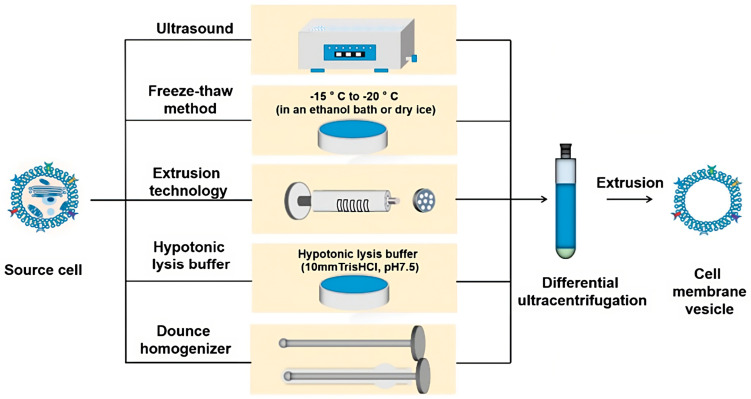
Different methods for obtaining cell membranes, licensed under a Creative Commons Attribution 3.0 unported license, adapted with permission from [142].

The process of the formulation of biomimetic NPs is conducted in distinct phases. According to the function and design of the intended NPs, the cell membrane should first be obturated by a suitable source before it can be created (Figure 13).

For example, cell lines or bacterial strains can be grown on a small scale in a laboratory setting, which is typically suitable for preclinical research. Suspension cells can be grown in shaker or spinner flasks, making harvesting easier than is the case for adherent cells, which need enzymatic or physical detachment. Furthermore, in vitro techniques for culturing engineered red blood cells (RBCs) or platelets have been reported, and such methods could potentially be used to produce future cell-based NPs [143,144,145]. The next step is to derive the membrane material, which is undoubtedly a simpler process for anucleate cells than nucleate ones. This could be obtained via cell lysis, which can be accomplished by sonication, mechanical homogenization, or centrifugation and final purification [146]. The abovementioned processes are followed by the fusion or coating of cell material with NPs.

In conclusion, the synthesis and production of biomimetic NPs require a complicated, multistep process, and it can be challenging to predict and control the interaction as well as stability of their numerous components; however, improvements in the creation of biomimetic nanoparticles have been made using a variety of methods, including obtaining source cells and obtaining membrane materials. To overcome these obstacles and realize the full potential of biomimetic hybrid NPs as an effective therapeutic approach for the management of various diseases, more research and development efforts are required.

### 3.1. Conventional Methods of Producing Biomimetic Hybrid NPs

Through the use of methods such as extrusion and ultrasonic energy, the creation of biomimetic nanoparticles (NPs) has advanced significantly. Extrusion is a good method for samples that do not tolerate much disruption, as it was originally used to coat NPs with cell membranes; in this process, cell membrane vesicles and NP cores are repeatedly forced through a membrane with nanometer-sized pores.

An alternative technique that is suitable for laboratory-scale fabrication and requires less labor is the presentation of ultrasonic energy in a mixture of cell membranes and NP cores; in this way, diverse types of NPs are created with desired capabilities and directed at various targets [147].

Admittedly, the difficulty of producing NPs in sufficient quantities in a standardized and reproducible manner has hampered their successful translation to clinical applications. Despite recent protocols attempting to address several obstacles, such as retaining the biological complexity of cellular membranes on the carrier surface, the control of physical and chemical properties over the final product, customization, and consistency, a major challenge remains in the development of adequate protocols for increasing nanomaterial manufacturing [148].

The methods and techniques mentioned above to produce biomimetic NPs have critical issues, such as the possibility of membrane degradation or failure to properly reposition membrane components after extrusion.

### 3.2. The Microfluidic Technique Applied to the Production of Hybrid Biomimetic NPs

**Figure 14:**
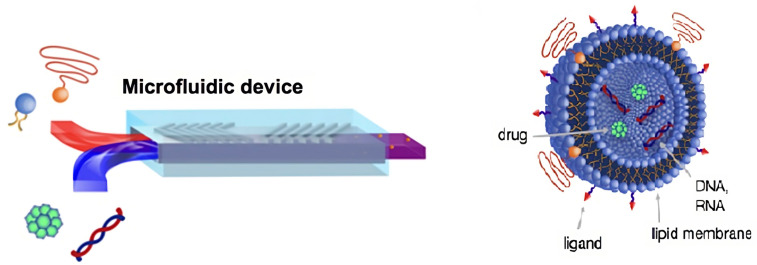
Microfluidic device illustration. Adapted and reproduced with permission from [150]. Copyright © 2023 Elsevier B.V.

To overcome all of the challenges regarding the production of nanoparticles as drug delivery systems (DDSs) (Figure 14), the microfluidic (MF) technique has influenced a wide range of applications, including biological analyses, chemical syntheses, single-cell analyses, tissue engineering, and ultimately NP production. MF applications provided several useful capabilities, including the ability to use extremely small quantities of samples and reagents to carry out the necessary separations and detections with high accuracy and precision, an inexpensive approach, shorter processing times, and small analytical device footprints [149].

NPs in MF systems are produced within small reaction channels with diameters between tens of and a few hundred microns. These systems have small channel dimensions that promote quick and uniform heat and mass transfer, as well as precise fluid control and manipulation, which lead to formulating NPs with better size distribution and overall improved NP yield. Various parameters can be adjusted to tune the final size and distribution of the resulting NPs, as well as their drug-loading capacity and batch-to-batch reproducibility. These parameters involve mixing properties, flow rate ratio (FRR), temperature, chip geometry, and the parameters relating to NPs as well as the materials used to create a particular type of NP, and we will briefly discuss each of them and how they affect the formulation of NPs through the MF technique.

In principle, the laws governing fluid flow at the macrometer and micrometer scales are the same. In microfluidic systems, the Reynolds number (Re) is typically lower than 100, indicating the omnipresence of the laminar flow and molecular-diffusion-dominated mixing. Altering the FRR changes the degree of mixing between the drug solution and nanoparticle precursor solution, resulting in various particle sizes and distributions. Increasing the FRR produces larger particles, whereas decreasing the FRR improves the mixing process and reduces particle size. Nonetheless, the flow ratio must be determined through experimental analyses to find out which one is most suitable for a particular NP formulation; therefore, a lot of other factors play a crucial role regarding which is the best flow rate to formulate biomimetic NPs. Mixing efficiency is another important parameter for creating homogenous NPs, and it is important to find a flow rate that promotes thorough mixing to obtain uniform NPs. The reaction kinetics, whether it is a chemical or biological reaction, also influences the FRR selection. The FRR facilitates the desired reaction kinetics, which allows sufficient time for the reaction to occur while preventing excessive residence time. Channel dimensions are another parameter that impacts the FRR; they determine the pressure, velocity, and mixing efficiency within channels, so the FRR should be altered to compensate for channel variations and optimize biomimetic NP production. Biomimetic hybrid NPs involve the incorporation of cell membrane components and large molecules into NPs, and it is crucial to preserve the integrity of these components. MF techniques can generate high shear force, which can lead to the deformation and damage of cell membranes or large biomolecules. To overcome this issue regarding shear stress, it is necessary to tune the microfluidic parameters to minimize the shear stress and reduce the potential for damage. Temperature is a crucial parameter in the process of the formulation of NPs and drug loading, and plays a crucial role; it affects the stability, solubility, diffusion rates, and reaction kinetics. By altering the temperature, one can control these parameters and therefore influence the properties of NPs. Another important parameter is the geometry of the chip, the size, and the shape of the channels in the microfluidic chip. Altering the width and height of microchannels impacts the residence time of fluids and hence affects the reaction kinetics and NP formation. Controlling the residence time, shear force, and mixing efficiency enables the precise manipulation of NP formulation through these systems. MF systems can be called up to achieve high-throughput production of coated NPs by parallelizing the MF channels or by using multiple MF devices operating simultaneously [151,152]. As explained above, the self-assembly of NPs by microfluidics can be achieved by using properly designed devices and differing the FRR between the aqueous and organic phases, the total flow rate (TFR) of the two streams, altering the ratio between the different ingredients used to formulate NPs and the temperature into the device during the production. It is possible to tune the final size and distribution of the resulting NPs, as well as their drug loading capacity and batch-to-batch reproducibility, by controlling the above parameters [153,154,155].

In an abovementioned study by Zinger et al., researchers examined the impact of adjusting the protein/lipid (P:L) ratio of leukocyte-based biomimetic nanoparticles (NPs) produced by the MF technique. Increasing the protein content had no impact on the size and polydispersity index (PDI) or on the concentration of NPs, whereas decreasing this ratio lead to a decrease in the surface charge (ZP), indicating that proteins have successfully been incorporated into the lipid bilayer. This represents an example of the tunability of different compounds used to produce biomimetic nanoparticles with the MF technique. There are many studies in which the microfluidic technique has been proven to be an excellent choice in the formulation of NPs, including biomimetic ones [156,157,158,159].

**Figure 15:**
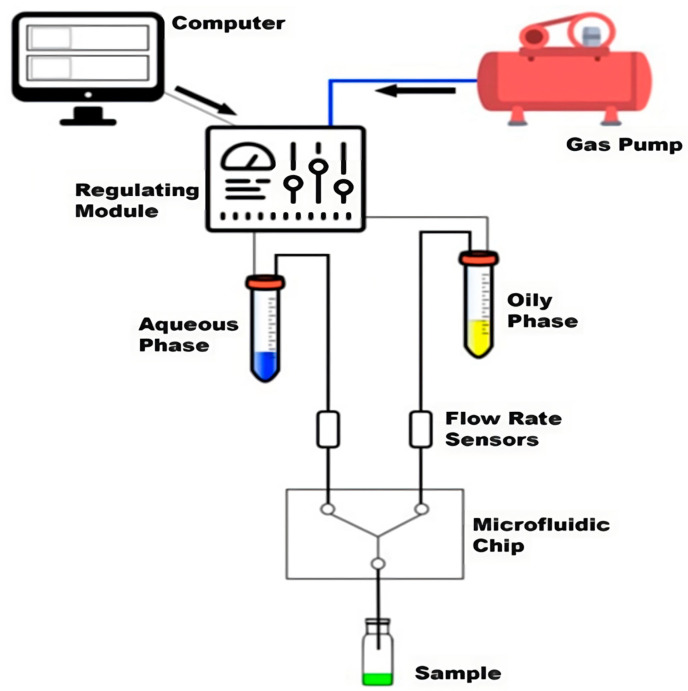
Schematic presentation of a microfluidic system and its components, licensed under a Creative Commons Attribution 3.0 unported license, adapted with permission from [163].

NanoAssemblr (NA) is a microfluidic-based system that has been recently created for the controlled, tunable, low-cost, and scalable manufacture of NPs, which is a system used for the formulation of NPs, including biomimetic ones [160,161,162] Researchers used NA in a study to create nanovesicles by incorporating membrane proteins from leukocytes into the lipid bilayer of liposome-like nanovesicles. NA involves the use of herringbone mixers and a microfluidic mixing chamber with a Y-shaped inlet channel. The herringbone micromixer of this microfluidic system causes the fluid stream in the channels to stream and fold Under laminar flow conditions, these systems, along with the herringbone structures on the channel floor, allow better mass transfer. These characteristics of NA enable fluid mixing, which allows NPs of desired properties to be formed. Researchers initially adjusted the mixing protocols; they determined the best FRR, total flow rate (TFR), and temperature by varying the aqueous and organic phase ratio through multiple tests, and concluded that the best conditions are 2:1 FRR, 1 mL/min TFR, and a temperature of 45 °C (Figure 15). Biomimetic NPs produced under these settings exhibited a diameter of 188 nm, PDI of 0,13, and surface charge of −12 mV. The biomimetic formulations showed size homogeneity, which was confirmed via a cryoEM analysis, and the production of these NA formulated leukosomes (Na-Leuko) demonstrated high reproducibility across multiple batches, highlighting the performance of production through an MF system. These findings show the potential of microfluidics in developing biomimetic nanovesicles with applications in biomedicine and other fields [37].

Another study discovered that microfluidic electroporation is a promising method for producing core–shell NPs. These biomimetic NPs were created using Fe_3_O_4_ magnetic NPs and RBC-membrane-derived vesicles. The researchers then evaluated the in vivo performance of RBC magnetic NPs (RBC-MNPs) and discovered that they performed significantly better than those made by conventional extrusion. RBC-MNPs produced using the microfluidic electroporation strategy have several advantages. The magnetic nanoparticle cores have superior magnetic and photothermal properties, whereas the RBC membrane shells possess long blood circulation properties. Furthermore, when compared to conventional extrusion, the complete cell membrane coating achieved by microfluidic electroporation ensures better treatment efficacy. According to this study, MF electroporation facilitates a better entry of MN NPs into RBC vesicles; the resulting biomimetic NPs presented better colloidal stability, which refers to the ability of produced NPs to remain evenly dispersed in a solution without aggregation compared with the biomimetic NPs produced by conventional extrusion. MF electroporation allows for precise flow control over the NPs and the membrane dispersions. This control ensures laminar flow conditions and prevents the aggregation of nanoparticles while enabling uniform coating. The facilitated efficient mixing the MF devices offer plays a great role in enhancing the interactions between NPs and cellular membranes, which also enables better coating properties and therefore enhanced effectiveness, as provided by in vivo studies conducted in the research [164]. MF methods are more efficient than conventional bulk mixing techniques for coating nanoparticles with cellular membranes. The ability of these systems to control flow rate, mixing properties, and interfacial interactions is crucial in the creation of biomimetic nanoparticles.

Liu et al. presented a work in which they created exosome membrane-PLGA NPs (EM-PLGA NPs) and cancer cell membrane-PLGA NPs (CCM-PLGA NPs) encapsulated with imaging agents using microfluidic sonication in a single step. The targeting efficacy of tumor-cell-derived EM-coated PLGA NPs was then compared to CCM-PLGA NPs in both in vitro and in vivo models. They discovered that EM-PLGA NPs exhibit superior homotypic targeting due to lower uptake by macrophages and peripheral blood monocytes, allowing for the immune-evasion-mediated targeting of homologous tumors. Overall, the findings indicate that microfluidic sonication is a promising method for producing biomimetic NPs with improved biocompatibility and targeting efficacy [165]. In another study, in which biomimetic NPs were fabricated with microfluidic technique, researchers used engineered high-density lipoprotein-mimetic NPs (eHNPs) to effectively deliver a Sonic Hedgehog signaling inhibitor (LDE225) to the cancer-stem-like cell population in SHH medulloblastoma cells (SHH MB). Through the scavenger receptor class B type 1 (SR-B1) and CD15, the eHNPs were designed to cross the blood–brain barrier (BBB) and target SHH MB cells. In vitro, ex vivo, and in vivo, the researchers demonstrated that eHNP-A1-CD15-LDE225 had dual-targeted delivery and an enhanced therapeutic effect on SHH MB cells. Microfluidic technology for NP formulation in this study enabled the efficient control of nanomaterial formation and characteristics, resulting in a narrow size distribution and high batch-to-batch replicability [166]. Microfluidics has a bright future because it has shown great promise in the fields of materials science and nanotechnology. Biomimetic NPs or drug carriers could be created using microfluidic synthesis for specific patients. This would allow for more precise and effective drug delivery, resulting in fewer side effects and better treatment outcomes (Table 3). The industrial-scale production of NPs remains a challenge, but microfluidic technologies offer promising possibilities for the creation of drug delivery systems that can be designed in an easy, cost-effective, and reproducible manner [167].

Even though this technology is still in its early stages and has some technical complexities, its potential for producing biomimetic NPs is enormous. Microfluidic synthesis, with further advancements and refinements, holds great promise as a strategy for the development of next-generation biomimetic NPs.

## 4. Conclusions

In conclusion, biomimetic NPs have shown promise in the creation of efficient drug carriers that can overcome the drawbacks of conventional routes of therapy. Improved drug delivery, decreased toxicity, and increased targeting specificity have all been demonstrated with the use of biomimetic NPs, such as those coated and fused with natural cell membranes; however, it is still difficult to apply these encouraging results from in vitro to in vivo systems, and more research is needed to fully comprehend the intricate interactions between these biomimetic NPs and the human body. In this review, particular attention was paid to several examples of hybrid biomimetic NPs, both coated and fused, to manufacturing procedures, and primarily to the advantages of this groundbreaking strategy for drug delivery systems. The processes of coating and fusing NPs involve the combination of synthetic NPs with natural cell membranes to produce biomimetic NPs with surfaces that mimic properties crucial for biointerfacing. This trend of producing nanocarriers that combine synthetic and organic NPs has been applied to several cell membranes, including those of cancer cells, red and white blood cells, cell membrane vesicles, and exosomes. Biomimetic hybrid NPs have demonstrated potential for improved therapeutic efficacy and targeted drug delivery; however, these nanocarriers’ creation and stabilization present challenges. Hybrid systems have demonstrated superiority over non-hybrid systems in terms of loading effectiveness, release kinetics, cellular uptake, and cytotoxicity. We also covered microfluidic technology as a novel method for manufacturing biomimetic NPs, with numerous advantages over conventional methods. Microfluidic systems’ precise fluid control and manipulation enable the development of uniform NPs with customized properties, which render them ideal for a wide range of applications in nanotechnology. The studies looked over in this review clearly demonstrated the potential of microfluidics in the production of biomimetic NPs and drug delivery systems, pointing out its ability to improve the functionality and performance of such systems. Despite challenges related to technical complexity and equipment costs, microfluidic synthesis offers a promising strategy for producing highly reproducible and customizable materials with reduced waste and increased efficiency.

## Figures and Tables

**Table 1:** Drugs encapsulated in exosomes and their therapeutic effect.

Drug	Disease	Therapeutic Effect	Reference
Paclitaxel	Prostate cancer	Increased drug cytotoxicity to cancer cells	[125]
Doxorubicin	Breast cancer	Enhanced drug delivery to the tumor site and inhibited tumor growth	[126]
siRNA	Alzheimer’s disease	Specific siRNA delivery to the brain	[127]
miRNA	Ischemic kidney injury	Protection of kidney function and reduced kidney injury	[128]
Curcumin	Lipopolysaccharide-induced shock	Increased anti-inflammatory activity	[129]
Dopamine	Parkinson’s disease	Increased therapeutic effect due to brain-specific drug delivery	[130]

**Table 3:** Examples of biomimetic NPs produced by the microfluidic technique and their advantages.

Biomimetic NPs	Advantages of MF Production	Reference
Leukocyte NPs	Reproducible and enhanced biomimetic NPs	[92]
Leukocyte–liposome NPs	Versatile, reproducible, robust, and efficient	[37]
Fe_3_O_4_–RBC NPs	Better performance in vivo in comparison with conventional extrusion	[164]
Exosome–PLGA NPs and cancer cell membranes	Improved biocompatibility and targeting efficiency of NPs	[165]
eHNPs to deliver SHH inhibitor (LDE 225)	Narrow size distribution and high batch-to-batch reproducibility	[166]
